# Editorial: Extracellular Vesicles in CNS Diseases

**DOI:** 10.3389/fmolb.2022.943369

**Published:** 2022-06-09

**Authors:** Raffaella Soleti, Maria Antonietta Panaro, Tarek Benameur, Giovanni Messina, Chiara Porro

**Affiliations:** ^1^ Univ Angers, Nantes Université, Inserm, CNRS, CRCI2NA, SFR ICAT, Angers, France; ^2^ Department of Biosciences, Biotechnologies and Biopharmaceutics, University of Bari, Bari, Italy; ^3^ Department of Biomedical Sciences, College of Medicine, King Faisal University, Al-Ahsa, Saudi Arabia; ^4^ Department of Clinical and Experimental Medicine, University of Foggia, Foggia, Italy

**Keywords:** extracellular vesicles, CNS diseases, amyotrophic lateral sclerosis, miRNA - microRNA, exosomes

Extracellular vesicles (EVs) are considered as a new tool for cell to-cell communication in the brain, in both brain health and diseases. Under physiological and pathological conditions, all cells release EVs. Based on their biogenesis and size, EVs can be divided into three categories: exosomes, micro-vesicles, and apoptotic bodies. Many body fluids contain EVs, which enclose membrane, cytosolic membrane, and nuclear components such as proteins, lipids, and a variety of RNAs, including microRNAs, other non-coding RNA species with regulatory properties, as well as DNA fragments. EVs carry a variety of bioactive molecules and act as inter- and intra-cellular messenger, representing the fingerprints of their originating cells. EVs have the potential to influence target cell fate, inducing significant changes that may lead to their contribution to different pathophysiological processes. In the central nervous system (CNS) diseases, EVs and their content have emerged as potential biomarkers for diagnosis and prognosis (Panaro et al., 2020; Benameur et al., 2022).

The purpose of the present Research Topic (RT) is to bring together recent findings in the in the field of EVs in the brain. EVs have been studied for their role in cellular communication, but many questions remain unanswered, including those about EVs biogenesis, cargo characterization, and the role of EVs in the brain, with particular focus on their contribution to the progression of neurodegenerative diseases.

A better understanding of the pleiotropic role of EVs in brain homeostasis could help researchers to gain the knowledge they need to develop new therapeutic strategies to treat and reverse neurological diseases.

A total of 26 high-ranking international researchers and scientists have contributed to a compilation of four articles in the current Research Topic. Among the four accepted articles, readers will find one original research article (Yamaguchi et al.), two reviews (Chen et al.; Wang et al.) and one mini review (Nakamya et al.).

Modifications of protein cargo and nucleic acid from exosomes isolated from human biological fluids have been linked to several pathologies, including amyotrophic lateral sclerosis (ALS). ALS is a serious neurodegenerative disease characterized by a progressive motor neuron degeneration (MN) that results in muscle control loss. The majority of patients die of respiratory complications within 3–5 years of diagnosis with ALS (Cleveland and Rothstein, 2001; Chiò et al., 2009; Brown and Al-Chalabi, 2017; Hosaka et al., 2019; Roy et al., 2019). Regrettably, the molecular pathways involved in motor neuron degeneration remain unknown. Indeed, the research for useful diagnostic biomarkers and treatment strategies is difficult, despite the fact that genetic and non-genetic causes have been proposed. Exosomes have been identified as promising candidates for studying ALS pathogenesis, as they can act as biomarkers for disease diagnostic and progression. In their review, Chen et al. revisited a summary of existing knowledge on exosome the biogenesis and integration, as well as their function and heterogeneity in the brain. They demonstrated how ALS-related proteins are secreted and propagate through exosomes, probably contributing to progressive disease dissemination throughout the body. They also illustrated several extracellular miRNAs associated with the onset and progression of ALS, as well as the therapeutic potentials of exosomes, despite the lack of standardized methodology for exosomes detection and isolation. Wang et al. focused their review on the use of EVs as therapeutic strategies for ALS. Exosomes can cross the Blood Brain Barrier (Ediriweera et al., 2021), this characteristic making them a potential candidate for non-cell therapy in neurodegenerative diseases, such as ALS (Ciregia et al., 2017). EVs can be isolated from a variety of cells of different origins. Mesenchymal stem cells have the capacity to release a higher amount of EVs that have beneficial effects on their target cells. Indeed, the authors demonstrated promising therapeutic strategies against ALS using of EVs derived from adipose-derived stem cells and human bone marrow-derived endothelial progenitor cells.

The authors then discussed the use of EVs as drug delivery tool for ALS. Chemical, biological, or physical methods can be used to enrich EVs with therapeutic drugs or bioeffectors (Bonafede et al., 2016). Finally, authors explored other interesting tools as potential therapeutics: plant-derived EVs and exosome-mimics, either unmodified or engineered. The innovative research on EVs represents an encouraging strategy for the treatment of ALS.

Yamaguchi et al. elegantly investigate the role of EVs in Hereditary Transthyretin Amyloidosis (ATTRy), a disease in which insoluble proteins, known as amyloids, are deposited in various organs, causing multiple damage. They showed that cell-derived EVs were involved in the formation of transthyretin (TTR) amyloid deposits on the membrane of small EVs and cells. Similarly, human serum EVs altered the intensity of TTR aggregation and deposition, resulting in lower TTR in serum-derived EVs from ATTR patient. The increased amount of EVs in ATTR patients suggested that they actively contribute to enhance ATTR amyloidosis and metabolism. As a result, serum-derived small EVs offer an opportunity to develop diagnostic and therapeutic tools for patients with ATTR amyloidosis.

To complete the analysis of the role of EVs in the CNS, Nakamya et al. present a synthesis of current knowledge about extracellular vesicles carrying mitochondrial components, with a focus on several neurological disorders of the CNS such as Parkinson’s Disease, Down Syndrome, Alzheimer’s Diseases and aging. In their minireview the authors also discuss the current findings that highlight cross talk between mitophagy and the EVs that carry mitochondrial cargo. The release of mitochondria-derived vesicles (MDVs), a subtype of EVs containing mitochondrial DNA and proteins, is triggered by mitochondrial dysfunction due to dysregulated mitophagy and the induction of oxidative stress.

The MDV content could be disseminated. Consequently, MDVs contribute to disease pathogenesis and progression. MDVs, on the other hand, aid in the preservation of cell functions by promoting the elimination of their content in lysosomal compartments. MDVs, according to the authors, could be used as biomarkers or as tolls in the development of novel therapeutic strategies for CNS disorders. Indeed, it is critical to develop research projects to broaden and standardize methodology for EVs studies, as well as to further investigate the functions and mechanisms involved in CNS disorders. Altogether, this collection of reviews and original researches for this Research Topic provides new perspectives and insights into the diverse links between EVs and their content, as well as their potential value as biomarkers of diagnosis and prognosis in CNS diseases.
